# Progress in Surgery of the Intestines

**Published:** 1903-09-26

**Authors:** 


### PROGRESS IN SURGERY OF THE INTESTINES.

(*Concluded from page* 437.)

**Ulceration in Typhoid Fever.**—McCrae and Mitchell[8] summarise their two years' experience as follows:—Two hundred and seventy-five cases of typhoid fever were treated and perforation of the intestine occurred eight times. Of these seven were operated upon with two recoveries, a third dying of toxæmia after a week. All of these seven were recognised within nine hours, except two, in which hæmorrhage from the bowel accompanied the perforation. In one case operation was not advised because the patient was evidently in extremis. Exploratory laparotomy was done in two cases in which no perforation was found. In one the symptoms proved to be due to intestinal hæmorrhage; in the other to a low grade of peritonitis. The first died; the second recovered. Eleven cases with suspicious abdominal symptoms were not operated upon. Of these two died and the autopsies showed no perforation. Shepherd[9] reports three successful cases. He has always made use of the lateral incision, and has usually found the perforation near the ileo-cæcal valve; by this incision the site of the perforation is more easily found than by the median incision, and with less disturbance to the parts and less moving about of the intestines and, consequently, with much less chance of general infection of the peritoneal cavity. Bowlby[10] also records a second case of successful operation for perforation in typhoid fever.

**Malignant Disease of the Colon.**—Littlewood[11] records 14 cases of colectomy with 10 recoveries. If there are not definite symptoms of obstruction at the time of operation, colectomy with end-to-end union is done. The divided portion is cleansed, and from both ends a circular portion of mucous membrane, from one-eighth to a quarter of an inch in breadth, is removed in order to have a good broad surface of the other coats to bring into apposition. Suturing is commenced at both cut edges at the mesenteric attachment, and the sero-muscular coats are united for half the circumference by means of catgut. The mucous membrane is now sutured around the whole circumference with the same material, the suture being interrupted by knotting at two or three points; now the remaining half of the sero-muscular coats is united by means of the first suture interrupted by knotting up two or three points of the circumference, so as to prevent the continuous stitch from making the lumen too narrow. The opening in the mesentery is then closed. If there is complete intestinal obstruction at the time of operation the bowel is opened at the first operation and the growth removed subsequently. Leech[12] suggests that if a patient of the cancer age with intestinal symptoms associated with wasting does not yield to treatment in a few weeks an exploratory laparotomy should be performed. James Swain[13] also records successful cases of colectomy.

**Chronic Colitis.**—Gibson[14] discusses a new measure for the treatment of colitis of chronic and aggravated type. He attains the desired result by establishing a fistula in the cæcum, whose functions are limited to the introduction of cleansing and healing agents, whilst not in any way giving exit to the contents of the bowel. This he does by reproducing in the cæcum the exact conditions of Kader's gastrostomy in which a valvular fistula is created by burying a tube in an inverted funnel of the stomach wall. In a few days the fistula becomes permanent and the tube is withdrawn, and is only re-introduced from time to time for conveying fluids into the stomach. In the intervals the patient can pursue his usual life, unhampered by the wearing of any apparatus or dressing; and the author shows that the cæcum lends itself to the creation of the fistula fully as well as does the stomach, and with the like convenience and comfort to the patient. The colon is irrigated through the catheter with nitrate of silver solution followed by normal saline solution. This is repeated at not longer intervals than twelve hours at first. The patient should be on a chiefly proteid diet. The tube remains *in situ* for a week or ten days. Closure of the fistula occurs spontaneously with the discontinuance of the daily passage of the catheter. Weir[15] instead of the above operation, makes use of the appendix vermiformis. He separates and draws this up into the abdominal wound, and fixes it there. The distal portion is then cut off, and the tube passed along the lumen of the appendix into the cæcum. No leakage of fæces occurs on removal of the tube.

[8] Med. Rec., New York, Feb. 14, 1903, p. 256. [9] Brit. Med. Jour., March 14, 1903, Epitome No. 175. [10] Lancet, Jan. 10, 1903, p. 91. [11] Lancet, May 30, 1903, p. 1514. [12] Quart. Med. Jour., Feb., 1903, p. 106. [13] Brit. Med. Jour., Jan. 10, 1903, p. 68. [14] Brit. Med. Jour., Dec. 6, 1902, Epitome No. 340. [15] Med. Chron., Nov., 1902, p. 135.

